# Emergent structured transition from variation to repetition in a biologically-plausible model of learning in basal ganglia

**DOI:** 10.3389/fpsyg.2014.00091

**Published:** 2014-02-11

**Authors:** Ashvin Shah, Kevin N. Gurney

**Affiliations:** Department of Psychology, University of SheffieldSheffield, UK

**Keywords:** action discovery, reinforcement, basal ganglia, variation, repetition

## Abstract

Often, when animals encounter an unexpected sensory event, they transition from executing a variety of movements to repeating the movement(s) that may have caused the event. According to a recent theory of action discovery (Redgrave and Gurney, 2006), repetition allows the animal to represent those movements, and the outcome, as an action for later recruitment. The transition from variation to repetition often follows a non-random, structured, pattern. While the structure of the pattern can be explained by sophisticated cognitive mechanisms, simpler mechanisms based on dopaminergic modulation of basal ganglia (BG) activity are thought to underlie action discovery (Redgrave and Gurney, 2006). In this paper we ask the question: can simple BG-mediated mechanisms account for a structured transition from variation to repetition, or are more sophisticated cognitive mechanisms always necessary? To address this question, we present a computational model of BG-mediated biasing of behavior. In our model, unlike most other models of BG function, the BG biases behavior through modulation of cortical response to excitation; many possible movements are represented by the cortical area; and excitation to the cortical area is topographically-organized. We subject the model to simple reaching tasks, inspired by behavioral studies, in which a location to which to reach must be selected. Locations within a target area elicit a reinforcement signal. A structured transition from variation to repetition emerges from simple BG-mediated biasing of cortical response to excitation. We show how the structured pattern influences behavior in simple and complicated tasks. We also present analyses that describe the structured transition from variation to repetition due to BG-mediated biasing and from biasing that would be expected from a type of cognitive biasing, allowing us to compare behavior resulting from these types of biasing and make connections with future behavioral experiments.

## 1. Introduction

Animals are capable of executing a huge variety of movements but, importantly, they can discover the specific movements that affect the environment in predictable ways and represent them as *actions* for later recruitment. Redgrave, Gurney, and colleagues have suggested that this occurs through a process they refer to as *action discovery* (Redgrave and Gurney, 2006; Redgrave et al., 2008, 2011, 2013; Gurney et al., 2013). Action discovery begins when an animal is executing movements within some context and an unexpected salient sensory event (such as a light flash) occurs. The unexpected sensory event causes a short-latency phasic increase in dopamine (DA) neuron activity (henceforth referred to simply as *DA activity*). Through its influence on the basal ganglia (BG)—a group of interconnected subcortical structures which, in turn, influence cortical activity—the increase in DA activity can help bias the animal to repeat the movements that preceded the unexpected sensory event under the same contextual circumstances. This *repetition bias* (Redgrave and Gurney, 2006) allows associative networks in the brain to learn and encode the movements as an action because it causes a frequent and reliable presentation of context, movements, and the sensory event as the outcome of those movements.

This transition from executing a variety of movements to repeating just one or a subset of movements often follows a non-random, structured, pattern. For example, consider a spatial task such that reaching to a specific location results in the outcome. Here, one type of structured transition from variation to repetition occurs if the animal gradually refines its movements so that movements that are further from the location decrease in frequency earlier than movements that are closer to the location.

The non-random structure of the transition from variation to repetition can be explained with “intelligent” or sophisticated cognitive mechanisms, e.g., by using an estimation of the range of movements that cause the outcome that gets more and more precise with repeated occurrences of the outcome. Similarly, other types of a structured transition may rely on other sophisticated notions such as optimality or uncertainty (e.g., Dearden et al. 1998; Dimitrakakis 2006; Simsek and Barto 2006). However, the process of action discovery is thought to be mediated primarily by simpler mechanisms involving DA modulation of the BG, and not sophisticated cognitive mechanisms. In this paper we ask the question, can *simple* BG-mediated mechanisms guide a structured transition from variation to repetition, or must sophisticated cognitive mechanisms always be recruited? To address this question, we present a computational model of BG-mediated biasing of behavior.

Our model will necessarily deal with a specific and, therefore, limited example of action discovery and so to establish its status, we now outline the model's wider context comprising various broad categories of action. For example, one type of action might involve making a particular gesture with the hand (as in sign language or hand signaling), regardless of the precise spatial location of the hand, and no environmental object is targeted. Another type of action involves manipulating objects in the environment (such as flipping a light switch or typing out a password). In this instance, space is weakly implicit (the objects are located somewhere); the key feature is the target object identity and its manipulation. In this paper, we focus on an explicitly spatial task: the relatively simple action of moving an end-effector to a particular spatial location. In the model task, a movement end-point to which to move must be selected. End-points that correspond to a target location elicit a reinforcement signal, and, importantly, reinforcement is not contingent on movement trajectory. The model task is inspired by behavioral counterparts we have used to study action discovery in which participants manipulate a joystick to find an invisible target area in the workspace (Stafford et al., 2012, 2013; Thirkettle et al., 2013a,b). While there may be “gestural” aspects of action in the behavioral task, in the model we ignore these and focus only on the spatial location of movement end-point.

In the next few paragraphs, we describe features of neural processing which our model incorporates that many other models of the BG do not. Biological theories of BG function suggest that the BG bias behavior not through direct excitation of their efferent targets, but, rather, through the selective relaxation of inhibition (i.e., disinhibition) of their efferent targets (Chevalier and Deniau, 1990; Mink, 1996; Redgrave et al., 2011). When the BG are presented with multiple signals, each representing an action or movement, these signals will have different activity levels signifying the urgency or *salience* of the “action request.” BG are supposed to process each signal through a neural population or *channel*, and inter-channel connections facilitate competitive processes resulting in suppression of BG output (inhibition) on high salience channels and increased output on the low salience channels (Gurney et al., 2001a,b; Humphries and Gurney, 2002; Prescott et al., 2006). Many models of BG function focus on how the multiple signals presented to the BG are transformed to the activity of the BG's output nucleus. Action selection in these models is then based on the latter's activity (e.g., Gurney et al. 2001a,b, 2004; Joel et al. 2002; Daw et al. 2005; Shah and Barto 2009). However, one important feature of our model is that it also takes into account the pattern of excitation from other areas to the BG's efferent targets (see also Humphries and Gurney 2002; Cohen and Frank 2009; Baldassarre et al. 2013). Thus, behavior results from BG modulation of their efferent target's response to excitation patterns, and is not just a mirror of the activity of the BG's output nucleus.

Further, many models of BG function focus on how the BG select from a small number of abstract independent behaviors (e.g., Gurney et al. 2001b; Daw et al. 2005; Cohen and Frank 2009; Shah and Barto 2009). While such representations may be appropriate for some behavioral tasks in experimental psychology, in ethological action discovery, the space of activities from which to select may be larger and adhere to some inherent topology. In our model, candidate locations to which to move are represented by a large number of topographically-organized neurons in cortex so that neighboring spatial locations are represented by neighboring neurons. Excitation to cortex follows a pattern in which all neurons are weakly excited initially, and that pattern evolves so that eventually only one neuron is excited strongly. This pattern is inspired by neural activity observed in perceptual decision-making tasks (Britten et al., 1992; Platt and Glimcher, 1999; Huk and Shadlen, 2005; Gold and Shadlen, 2007), and as suggested by evidence accumulation models of decision-making (Bogacz et al., 2006; Lepora et al., 2012).

We hypothesize that because the BG bias behavior by modulating cortical response to excitation, and that that excitation follows a structured pattern, simple BG-mediated biasing can result in a structured transition from variation to repetition in action discovery. Sophisticated cognitive mechanisms are not necessarily required to develop a structured transition.

In addition, behavioral biasing in action discovery is not thought to be driven by “extrinsic motivations” that are based on rewarding consequences and that dictate reinforcement in many types of operant conditioning tasks (Thorndike, 1911; Skinner, 1938) and computational reinforcement learning (RL) (Bertsekas and Tsitsiklis, 1996; Sutton and Barto, 1998). Rather, “intrinsic motivations” (Oudeyer and Kaplan, 2007; Baldassarre, 2011; Barto, 2013; Barto et al., 2013; Gottlieb et al., 2013; Gurney et al., 2013) that are triggered by the occurrence of an unexpected sensory event may drive DA activity and thus behavioral biasing in action discovery (Redgrave and Gurney, 2006; Redgrave et al., 2008, 2011, 2013; Gurney et al., 2013; Mirolli et al., 2013). In such cases, if the outcome does not represent or predict an extrinsically-rewarding event, reinforcement decreases as associative networks in the brain learn to predict its occurrence (Redgrave and Gurney, 2006; Redgrave et al., 2011). Rather than implement a model of prediction explicitly, we approximate its effects with a simple model of habituation in which the rate of reinforcement decreases as the target location is repeatedly hit (Marsland, 2009). This habituation model approximates the dependence of DA activity on outcome predictability in action discovery (Redgrave and Gurney, 2006; Redgrave et al., 2011), and is similar to that used in neural network models of novelty detection (Marsland, 2009).

In this paper, we use computational models to demonstrate that simple BG-mediated mechanisms can bias behavior, via their modulation of cortical response to a pattern of excitation, such that the transition from variation to repetition follows a structured pattern. We describe this structured pattern and show how it, along with the effects of habituation, lead to behavioral patterns in tasks in which one target area delivers a reinforcement signal, two target areas deliver reinforcement, or the target area that delivers reinforcement changes location. These experiments lead to predictions as to the type of behavior that would be expected when only simple BG-mediated mechanisms, and not more sophisticated cognitive mechanisms, bias behavior. We also run models that mimic a simple form of transition from variation to repetition that would be expected under sophisticated cognitive mechanisms by subsuming the effects of those mechanisms in a phenomenological way. In order to make contact with future behavioral experiments, we develop a novel characterization of behavioral trends which links these trends to underlying neural mechanisms that dictate different forms of biasing.

## 2. Methods

We use a computational model, based on established models (Gurney et al., 2001a,b; Humphries and Gurney, 2002), to control movement selection in a task that simulates reaching or pointing to specific target spatial locations. We provide here a conceptual overview of its mechanics; detailed equations are provided in the Supplementary section.

The model is a neural network model with leaky-integrator neuron units (henceforth referred to as “neurons” for brevity), the activities of which represent conglomerate neural firing rate of a group of neurons (Gurney et al., 2001a,b). Each brain area in the model, except for the area labeled “Context,” consists of 196 neurons spatially arranged in a 14 × 14 grid. Each neuron in each area is part of an “action channel” (Gurney et al., 2001a,b; Humphries and Gurney, 2002) such that its location in the grid corresponds to a movement toward the corresponding location of a two-dimensional workspace. For the purposes of this model, the workspace is of dimensions 14 × 14 units. Most projections from one area to another are one-to-one and not plastic; exceptions will be explicitly noted.

**Figure 1:**
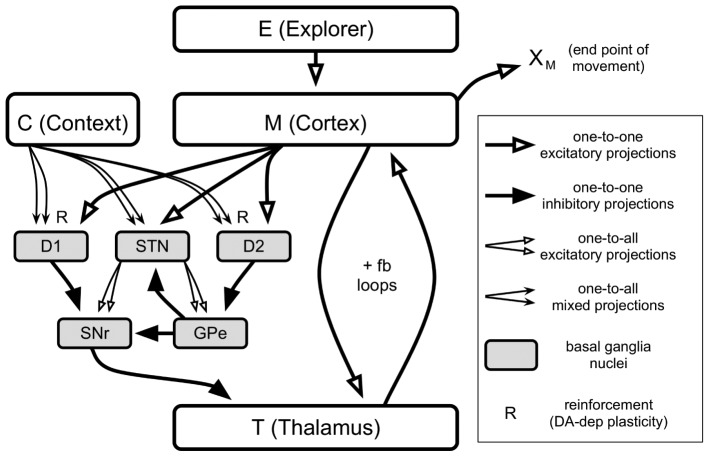
**Architecture of the model**. Each box except for “C (Context)” contains 196 neurons spatially arranged in a 14 × 14 grid. Context contains just one neuron. Types of projections are labeled in the legend on the right.

Figure 1 illustrates the gross architecture of the model. In brief, the end-point location of a movement, *X*_*M*_, is determined by the activities of neurons in “M (Cortex).” These neurons are excited by an exploratory mechanism, “E (Explorer),” and are engaged in positive feedback loops with neurons in “T (Thalamus).” The basal ganglia (BG, gray boxes) send inhibitory projections to Thalamus neurons, and they modulate the gain of the Cortex-Thalamus positive feedback loops (Chambers et al., 2011) through selective disinhibition of Thalamus neurons. Cortex and Thalamus represent grids of neurons that correspond to motor-related areas of cortex and thalamus, respectively.

### 2.1. Excitatory inputs to the neural network

There are two sources of excitatory input to the neural network.The first is labeled “C (Context)” and represents the context, such as participating in the current experiment. There is only one context for the results reported in this paper. Thus, Context consists of a single neuron with an output activity set to a constant value. Context influences BG activity through one-to-all projections to areas D1, D2, and STN. Projections to D1 and D2 are plastic and represent a context-dependent biasing of movements, as described in the subsection “Biasing of behavior.”

The second source of excitatory input is “E (Explorer),” which provides excitation to Cortex which, in turn, is responsible for movement. The Explorer is the source of variation required to explore the space of possible movements. This variation may be more or less random or structured according to the strategy used. However, these strategies are devised by other mechanisms, not explicitly modeled here, and we simply aim to capture the effects of such strategies in the Explorer.

In this paper, the Explorer is inspired by a range of experimental data. First, recordings in some areas of parietal cortices (Anderson and Buneo, 2002) show activation of neurons corresponding to a decision to make a movement that terminates at the location represented by those neurons. Further, several experimental studies, (Britten et al., 1992; Platt and Glimcher, 1999; Huk and Shadlen, 2005; Gold and Shadlen, 2007) show that neurons representing different decisions are weakly active early in the decision-making process. The activities of some neurons—corresponding to the executed decision in these experiments—increase at a greater rate than that of other neurons.

**Figure 2:**
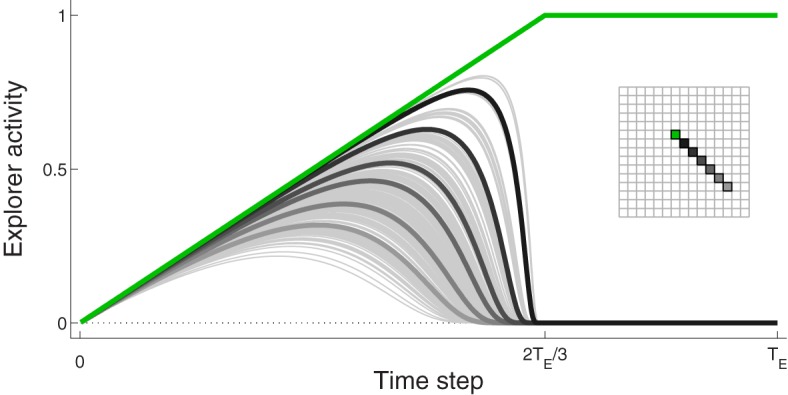
**Example of activity of Explorer neurons during a typical movement**. The activity of the neuron corresponding to the focus of excitation, *G*_exp_, is drawn in green. Selected neurons, colored in the inset, are drawn with thick lines in different shades of gray so as to demonstrate the spatial influence on excitation pattern. All other neurons are drawn in thin gray lines

We capture features of this behavior with a hand-crafted function describing, for a decision to move to a particular spatial location, the evolution of activity for every neuron in the Explorer. Early in the process, all neurons are weakly-excited with low activation levels. Neural activity evolves such that, as confidence in a particular movement increases, so does the corresponding neuron activity. The activities of other neurons increase to a lesser degree. An example of this behavior is shown in Figure 2; it is described in greater detail in the next paragraph and in the Supplementary section.

For each movement, a particular neuron in Explorer, labeled *G*_exp_, is chosen. If we suppose that sophisticated cognitive mechanisms are not devoted to movement selection, *G*_exp_ is chosen randomly. The activity of the neuron corresponding to *G*_exp_ increases linearly to one (green line in Figure 2). The activities of surrounding neurons change according to a Gaussian-like function centered at *G*_exp_. They first increase and then decrease; those furthest from *G*_exp_ increase by a small amount and then quickly decrease to zero, while those closer to *G*_exp_ increase by a larger amount and decrease at a later time point to zero. The pattern of activity such that the activity of neuron *G*_exp_ is one and the activities of all other neurons are at zero is held for brief time, and then the activities of all neurons are set to zero. This evolution takes *T*_*E*_ time steps, which is the number of time steps in a trial.

If, in contrast, we assume sophisticated cognitive mechanisms do influence movement selection, *G*_exp_ is chosen in order to reflect that strategy, e.g., according to some heuristic search such as a spiral pattern or quadrant-by-quadrant search. In this paper we examine behavior that results when cognitive mechanisms do not influence movement selection as well as behavior that results from a simple pattern, as described in the subsection “Biasing of behavior.”

### 2.2. Cortex and thalamus

“Cortex” represents cortical areas that encode high-level movement plans such as reaching or pointing to a location (Anderson and Buneo, 2002). In our model, the spatial location of a neuron in Cortex corresponds to a target spatial location in the workspace, or movement end-point, to which to reach. Cortex (M) receives excitatory projections from Explorer and Thalamus (T) which preserve channel identity; that is, the neurons representing a given channel in Explorer and Thalamus project to the corresponding neuron in Cortex. In turn, Thalamus receives channel-wise excitatory projections from Cortex, and channel-wise inhibitory projections from SNr (a nucleus of the BG called the substantia nigra pars reticulata). Cortex and Thalamus therefore form a positive feedback loop referred to as a *Cortex-Thalamus loop*, for each channel which is excited by the corresponding channel in Explorer. The gain of a Cortex-Thalamus loop is modulated by inhibitory projections from SNr neuron to Thalamus (Chambers et al., 2011). When the activity level of an SNr channel is low, the corresponding Thalamus neuron is said to be *disinhibited* and its Cortex-Thalamus loop has a high gain. A Cortex-Thalamus loop with a high gain is more easily-excited by the corresponding Explorer neuron.

### 2.3. Basal ganglia

The functional properties of BG architecture have been described in detail in prior work (Gurney et al., 2001a,b; Humphries and Gurney, 2002; Redgrave et al., 2011). Briefly, the BG is a subcortical group of brain areas with intrinsic architecture that is well-suited to select one behavioral option among competing options. The BG implement an off-center on-surround excitation pattern: The BG channel *i* that is most strongly-excited by its cortical “action request” inhibits the corresponding target channel (neuron) in Thalamus the least, while other Thalamus channels *j* ≠ *i* are further inhibited. Thus, Cortex-Thalamus loop *i* is most easily-excited by input from Explorer to Cortex, and other Cortex-Thalamus loops *j* ≠ *i* are harder to excite by input from Explorer to Cortex. These properties are similar in some ways to those of a winner-take-all network between the competing channels, but additional architectural features of the BG ensure better control of the balance between excitation and inhibition (Gurney et al., 2001a,b). D1 and D2 refer to different populations of neurons (named after the dopamine receptors they predominantly-express) in a nucleus of the BG called the striatum. The pathway comprising D1 and STN (subthalamic nucleus) performs the selection with an off-center on-surround network in which D1 supplies focussed (“central”) inhibition and the STN a diffuse (“surround”) excitation. The pathway through D2 regulates the selection by controlling, though GPe (external segment of the globus pallidus), the excitatory activity of STN (Gurney et al., 2001a,b).

### 2.4. From cortical activity to behavior

Movement in this model is a function of the activities of the Cortex neurons. Each neuron with an activation greater than a threshold η “votes” to move to the location represented by its grid location with a strength proportional to its activity (i.e., using a population code, Georgopoulos et al. 1982). In most cases, because of the selection properties of the BG, the activation of only one Cortex neuron rises above η. At each time step *t*, the target location to which to move, *X*_*M*_(*t*), is an average of the locations represented by Cortex neurons with activities above η, weighted by their activities. At each *t*, if any Cortex neuron is above η, a simple “motor plant” causes a movement from the current position (*x*_*p*_(*t*)) toward *X*_*M*_(*t*) (see Supplementary section for equations). Movement evaluation, and hence any learning, is based only on *x*_*p*_(*T*_*E*_), the position at time *T*_*E*_ (the last time step of a trial). Thus, end-point of movement, not movement trajectory, is evaluated in this model.

### 2.5. Biasing of behavior

Targets are circular areas within the workspace. A target is considered hit when ||*x*_*p*_(*T*_*E*_) − *X*_*G*_|| < θ_*G*_, where *X*_*G*_ is the location of the center of target *G* and θ_*G*_ (= 1.1) is the radius. Thus, a movement to the location represented by neuron *i* that corresponds to the center of the target, or to locations represented by the immediate four neighboring neurons, is within the target's radius. When a target is hit, behavior is biased so that the model is more likely to make movements to the target. This repetition bias (Redgrave and Gurney, 2006) can be implemented in two ways in this model.

The first way is “BG-mediated biasing,” which is based on dopamine-dependent plasticity at the corticostriatal synapses (Calabresi et al., 2007; Wickens, 2009), and is implemented as a Hebbian-like rule governing plasticity to weights onto striatal D1 and D2 neurons. When the end-point of movement is evaluated (at time *T*_*E*_ of a trial), usually only one neuron (*i*) in each of Cortex, D1, and D2 have an activity above zero. If the target is hit, the weights from Cortex neuron *i* to D1 neuron *i*, Cortex neuron *i* to D2 neuron *i*, the Context neuron to D1 neuron *i*, and the Context neuron to D2 neuron *i* are increased according to equations of the following form (see Supplementary section for full equations):

(1) $$\Delta w_{i}=\alpha \;\beta^{N_{k}-1}\;y_{pre}\;y_{post}\;(W_{max}-w_{i}),$$

where *w*_*i*_ is the weight, *y*_pre_ is the activity of the presynaptic neuron, *y*_post_ is the activity of the postsynaptic neuron, α is a step-size, *W*_max_ (= 1) is the maximum strength of a synapse, β (= 0.825) is a *habituation* term (Marsland, 2009), and *N*_*k*_ is the number of times target *k* has been hit. If the target is not hit, the weights are decreased. Weights from Cortex to striatum have a lower limit of zero, while weights from Context to striatum have a lower limit of −0.1. Neurons that have greater afferent weights are more-easily excited than are neurons with lower afferent weights.

Neurons in D1 and D2 that correspond to movements that were reinforced are excited by the Context neuron from the first time step of a trial onward, and neurons that correspond to movements that were not reinforced are weakly inhibited by the Context neuron. (We use negative weights to approximate the inhibitory effects of striatal interneurons, Koos and Tepper 1999; Bolam et al. 2006). Thus, weights from the Context neuron to D1 and D2 represent an *a priori* bias in favor of movements that were reinforced, and against movements that were not reinforced. This bias is context-dependent and, while there is only one context for the results reported in this paper, multiple contexts can be represented by multiple context neurons with similar learning rules. Neurons in D1 and D2 are also excited by Cortex neurons, which, early in a trial, are all weakly-excited by Explorer. Because the projections from Cortex to D1 and D2 are plastic, movements that were reinforced are more-easily excited by Cortex than movements that were not reinforced.

Thus, with BG-mediated biasing, channels corresponding to making a movement to locations that are within the target area are easily-excited by weak inputs from the Explorer after the target has been hit several times. Channels corresponding to movements that do not hit the target are made to be more difficult to excite.

The second way by which repetition bias is implemented in this model is referred to as “Cognitive biasing,” whereby *G*_exp_ is chosen according to some strategy or pattern. Under cognitive biasing in this paper, the set of neurons in Explorer from which *G*_exp_ is chosen corresponds to a spatial area, centered around the location of the target, that decreases in size each time the target is hit (we describe this pattern in detail in the Supplementary section). This is a simple hand-crafted form of biasing that mimics a decrease in variation and increase in repetition by “zooming in” on the target as the target is repeatedly hit. It is meant to capture the effects of behavioral biasing as mediated by “sophisticated cognitive” or “intelligent” mechanisms. If there is no Cognitive biasing, *G*_exp_ is randomly chosen as described earlier.

### 2.6. Model experiments

A model run consists of having the model select movements for 300 trials (where a trial consists of executing one movement). Movements were reinforced (Equation 1) when they hit a particular target. We examined behavior that results from reinforcing one target, two targets simultaneously, and one target and then another. The targets are referred to *G*_1_, *G*_2far_ (which is far from *G*_1_), and *G*_2near_ (which is near *G*_1_). Experiments 1 to 4 were conducted to describe patterns of behavior under simple, “non-intelligent,” BG-mediated biasing and different conditions of reinforcement. Experiment 5 was conducted to describe patterns of behavior under BG biasing, Cognitive biasing, and both.

- **Experiment 1: Single target** (*G*_1_): We ran 50 independent runs of 300 movements during which BG biasing (and not Cognitive biasing) was used to reinforce movements that hit *G*_1_.
- **Experiment 2: Two simultaneous targets** (*G*_1_ **and** *G*_2far_): We ran 50 independent runs of 300 movements during which BG biasing was used to reinforce movements that hit either *G*_1_ or *G*_2far_.
- **Experiment 3: Reinforce** *G*_1_, **then** *G*_2far_, **then** *G*_1_ **again**: We ran 50 independent runs of 900 movements during which BG biasing was used to reinforce movements that hit *G*_1_ for the first 300 movements, then to reinforce movements that hit *G*_2far_ (but not those that hit *G*_1_) for the next 300 movements, and then reinforce movements that hit *G*_1_ (but not those that hit *G*_2far_) for the final 300 movements.
- **Experiment 4: Reinforce** *G*_1_, **then either** *G*_2far_ **or** *G*_2near_: We ran 50 independent runs of 600 movements during which BG biasing was used to reinforce movements that hit *G*_1_ for the first 300 movements and then to reinforce *G*_2far_ (but not those that hit *G*_1_) for the next 300 movements. We ran another 50 independent runs of 600 movements during which BG biasing was used to reinforce movements that hit *G*_1_ for the first 300 movements and then to reinforce *G*_2near_ (but not those that hit *G*_1_) for the next 300 movements.
- **Experiment 5: Different bias conditions**: We ran 50 independent runs of 300 movements during which Cognitive biasing (and not BG biasing) was used to reinforce movements that hit *G*_1_. We ran another 50 independent runs of 300 movements during which both BG biasing and Cognitive biasing were used to reinforce movements that hit *G*_1_.

## 3. Results

### 3.1. Experiment 1: single target (*G*_*1*_)

Recall that there are two sources of excitation to the model, as explained in Methods section 2.1: the Context neuron, which projects to D1, D2, and STN; and the Explorer, which projects to Cortex (see also Figure 1). As described in Methods section 2.1, a focus of excitation, *G*_exp_, is chosen randomly, and the activities of neurons in the Explorer follow a hand-crafted pattern such that all neurons are weakly-excited initially, but that activity focuses so that only the neuron corresponding to *G*_exp_ is strongly-excited (see Figure 2). If the weights onto D1 and D2 remain at their initial values, Explorer activity will result in a movement made to the location represented by *G*_exp_.

**Figure 3:**
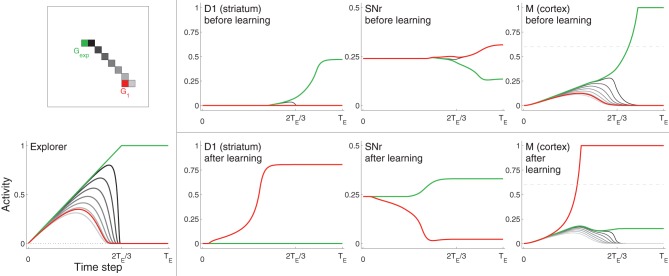
**Example neural activity for selected neurons from D1, SNr, and Cortex (M) at different points in training**. Neurons are colored according to their spatial location in the grid (**top left**). The red neuron corresponds to the center of target *G*_1_, the green neuron corresponds to the location of *G*_exp_ (focus of excitation in Explorer), which is not within the target area. Other neurons, most of which are located in between the *G*_1_ and *G*_exp_, are colored in gray (darker gray neurons are closer to *G*_exp_). **Bottom left**: activities of Explorer neurons. **Rightmost three graphs on top**: activities of neurons from D1, SNr, and M before learning (i.e., before the target was hit). Rightmost three graphs on bottom: activities of the same neurons after learning, i.e., after the target was hit several times. Note that the maximum of the vertical axis of SNr is 0.5, while that of the other graphs is one. Horizontal dashed line of graphs for M (right) represents η.

In Experiment 1, there was a single target, *G*_1_, located in the lower right area of the work space (center of target colored in red in the upper left graph in Figure 3). When the target was first hit, it was because the Explorer happened to choose a *G*_exp_ that was within θ_*G*_ of target center. As described in Methods section 2.5, when the target is hit, the corticostriatal weights that project to striatal neurons corresponding to the movement just made are increased (Equation 1). When a target is not hit, the weights decrease. The weight change influences how the BG modulates the gain between Thalamus and Cortex positive feedback loops (Methods sections 2.2 and 2.3), and hence how Cortex responds to excitation from Explorer.

#### Neural activity

Figure 3 shows selected neuron activity resulting from the same excitation from the Explorer during early movements (“before learning”) and during late movements (“after learning”). Excitation from Explorer is illustrated in the lower left graph, and the color scheme indicating which neuron's activity is plotted is illustrated in the upper left graph. In this example, activities of neurons corresponding movements made to *G*_exp_ are plotted in green; those corresponding to the center of the target (*G*_1_) are plotted in red; and those corresponding to a subset of neurons near or between *G*_exp_ and *G*_1_ are plotted in shades of gray. (Compare with Figure 2 and Methods section 2.1.) *G*_exp_ is not within the target area. The top row of graphs to the right of the color scheme graph plot neuron activity in striatum D1, neuron activity in SNr, and neuron activity in Cortex in the untrained model. As excitation from Explorer evolved over time, Cortex neurons increased accordingly due to the direct one-to-one projections from Explorer to Cortex and positive feedback loops with Thalamus (as described in Methods section 2.2). Cortex activity directly excited striatal neurons due to direct one-to-one projections to striatum D1 and striatum D2 (as described in Methods section 2.3). In this case, striatal neurons corresponding to *G*_exp_ increased in activity. Because no learning has occurred yet, Context did not bias activity in striatum as all projections from Context to striatum remained at zero. Intra-BG processing (described in Methods section 2.3) resulted in a decrease in activity of SNr neuron corresponding to *G*_exp_, and an increase in all other SNr neurons. This disinhibited the Thalamus neuron corresponding to *G*_exp_, increasing the gain on the positive feedback loop with Cortex neuron corresponding to *G*_exp_, thus allowing it to increase in activity even more. In addition, the increased activity of all other SNr neurons further decreased the positive feedback gain between other Cortex-Thalamus neuron pairs (Chambers et al., 2011). In this example, weights into D1 and D2 have not undergone any changes, i.e., the target has not been hit, so there is no biasing from Context. Thus, the BG facilitated the selection of the movement suggested by Explorer (move to location *G*_exp_) and inhibited the selection of other movements.

After the target had been hit many times, the weights from Context to striatal neurons D1 and D2, and from Cortex to D1 and D2, that correspond to movements made to a location within the target zone (in this example, the center of *G*_1_) increased (as described in Methods section 2.5 and Equation 1), and the weights to all others decreased by a small amount. Neuron activity in response to the same excitation from Explorer after learning is illustrated in the bottom, right most three graphs of Figure 3. Neurons that correspond to *G*_1_ (plotted in red) are referred to as *s*_*G*_. Because weights from Context to *s*_*G*_ in D1 and D2 have increased, the activity of neuron *s*_*G*_ in D1 and D2 increased faster due to excitation from Cortex than did that of other neurons, including that of neurons that correspond to movements made to *G*_exp_. This caused a decrease in the activity of SNr neuron *s*_*G*_ and an increase in the gain of the corresponding Cortex-Thalamus positive feedback loop (described in Methods section 2.2). Hence, the weak excitation to Cortex neuron *s*_*G*_ at the beginning of a movement period was sufficient to initiate a positive feedback process between the corresponding neuron *s*_*G*_ in Cortex and Thalamus, causing more excitation to neuron *s*_*G*_ in D1 and D2, even further disinhibition of the feedback loop, and further inhibition of the loops of other neurons. BG-mediated bias was in favor of movements toward *G*_1_, implemented by an increase in weights from Context and Cortex to the neurons in D1 and D2 that correspond to a movement to *G*_1_ (Equation 1). Thus, Cortex neuron *s*_*G*_ increased above η and movement was made to the location corresponding to *G*_1_, even though the Explorer more-strongly excited neurons corresponding to movements made to *G*_exp_.

#### Movement redistribution under contextual bias

The biasing of activity within the BG, BG's regulation of Cortex-Thalamus loop excitability, and the gradual focusing of excitation from Explorer to Cortex, comprise simple mechanisms that results in a seemingly “intelligent” structured transition from variability to repetition. After the target had been hit by chance a few times, weights from Context to neurons *s*_*G*_ in D1 and D2, and weights from neuron *s*_*G*_ in Cortex to neurons *s*_*G*_ in D1 and D2, were increased a little (Equation 1). When Explorer later chooses *G*_exp_ near *G*_1_, the resulting relatively high excitation to Cortex neuron *s*_*G*_, combined with the increased gain at Cortex-Thalamus loop *s*_*G*_ and decreased gain to other loops, excited Cortex neuron *s*_*G*_ while preventing other Cortex neurons from increasing past η. Thus, a movement to the target was made when Explorer chose *G*_exp_ near *G*_1_: the target was hit with an increased likelihood, and movements to areas near the target were made with a decreased likelihood. We refer to this pattern as a “bias zone,” centered at *G*_1_, that increases in size the more often the target is hit.

**Figure 4:**
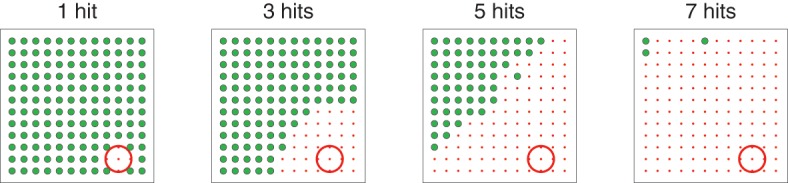
**Illustration of the “bias zone” effect. In each graph, the target was first hit *N* times (labeled at the top of the graph)**. Then, learning was turned off and movement for each possible value of *G*_exp_ was evaluated. Each dot represents the spatial location corresponding to *G*_exp_. Large green dots represent locations of *G*_exp_ that resulted in movements that hit the location corresponding to *G*_exp_. Small red dots represent locations of *G*_exp_ that, because of biasing implemented by weights onto D1 and D2, resulted in movements that hit the target (represented by the red circle in the lower right).

Figure 4 shows how the bias zone increases as the number of times the target has been hit increases. In order to produce this figure, the model was run with *G*_exp_ set to *G*_1_ for a set number of times. Then, learning was turned off and model response for *G*_exp_ set to each possible location was examined. Each graph in Figure 4 plots the location of *G*_exp_ in the workspace: green dots indicate locations of *G*_exp_ that result in movements made to those locations; red dots indicate locations of *G*_exp_ that result in movements made to locations within the target area (red circle). The title of each graph indicates how many times *G*_exp_ was set to *G*_1_ before response was examined. The expansion of the bias zone determines an “intelligent-looking” structured transition from variation to repetition in that it follows a non-random pattern.

**Figure 5:**
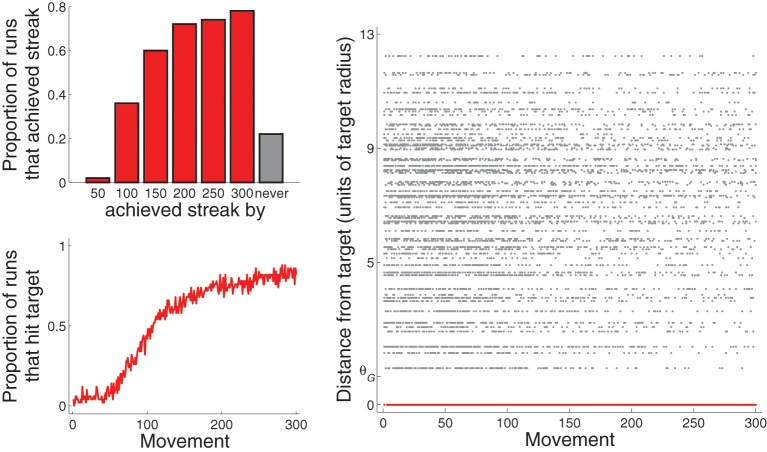
**Performance across all 50 runs for Experiment 1: A single target (and only BG biasing)**. **Top left**: proportion of runs that achieved streak of hitting target ten consecutive times by the movement 50, 100, 150, …, or 300. Note that the bar graphs are cumulative. **Bottom left**: proportion of runs that hit target as a function of movement number. **Right**: Distance from the center of the target (in units of target radius) of each movement from all 50 runs. That for movements that hit the target are drawn in red and are at value 0 of the vertical axis.

For the purposes of this paper, model behavior is considered to be well-learned when a “streak” of hitting the target with ten consecutive movements is achieved. Figure 5, top left, plots the proportion of 50 runs that achieved this streak by various points of experience. About 40% reached it by 100 movements, and almost 80% reached it by 300 movements. A little over 20% did not achieve it by 300 movements. Figure 5, bottom left, plots the proportion of 50 runs that hit the target as a function of movement number. The proportion reaches about 0.8 by movement number 300.

Figure 5, right, plots, for each movement across the 50 runs, the distance between the movement and *G*_1_ as a function of movement number. The distance of movements that hit *G*_1_ are plotted in red (and are all at zero). As movement number increases, the density of movements near *G*_1_ but that did not hit *G*_1_ decreases at a faster rate than the density of movements far from *G*_1_. This pattern is due to the expanding bias zone (Figure 4). We develop a method for quantifying this pattern in the section describing results of Experiment 5 (and in the Supplementary section). Experiment 4 describes behavior in a more complicated task that results from this pattern.

#### Effect of cortical noise on model performance

**Figure 6:**
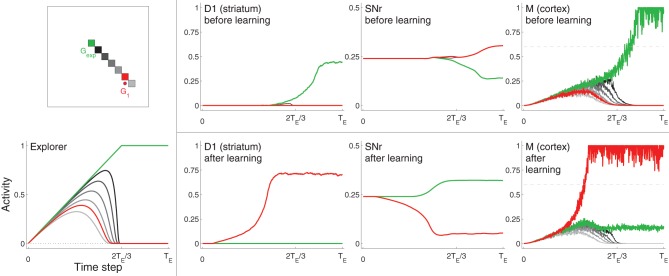
**Example neural activity for selected neurons from D1, SNr, and Cortex (M) at different points in training for a model with low levels of noise (**σ **= 0.1) added to Cortex (M) neuron activity (see text for details)**. This figure is plotted in a manner similar to that of Figure 3. Neurons are colored according to their spatial location in the grid (top left). The red neuron corresponds to the reinforced movement that hit target *G*_1_ in this example. Note, however, that, unlike with Figure 3, the reinforced movement is one unit away from the center of *G*_1_ (the center of *G*_1_ is marked with a closed red circle). (Recall that the radius of the target is 1.1 units, so movements made to the center of *G*_1_ or to the immediate neighbors of the center are reinforced.) The green neuron corresponds to the location of *G*_exp_ (focus of excitation in Explorer), which is not within the target area.

The capability of the model to bias movements toward *G*_1_ is due in part to the pattern of excitation from Explorer to Cortex (Figure 2), which weakly-excites all Cortex neurons by very similar amounts early in a trial. This suggests that model performance may be sensitive to unpredicted deviations from this pattern. To investigate this, we ran simulations in which signal-dependent noise (Harris and Wolpert, 1998) was added to Cortex neurons (which project to the BG and Thalamus, and from which movement is determined). In particular, at each time step: *y* ← [*y* + *y N*(0, σ)]^1^_0_, where *y* is the output activity of a Cortex neuron, *N*(0, σ) refers to a number drawn randomly from a zero-mean Gaussian distribution with standard deviation σ, and [x]^1^_0_ returns 0 if *x* < 0, 1 if *x* > 1, and *x* otherwise. The proportion of the last 30 movements of all runs under a particular noise condition that were made to *G*_1_ were 0.82, 0.64, 0.53, and 0.20 for σ levels of 0 (no noise), 0.1, 0.3, and 0.5, respectively. Thus, the model was able to learn to repeatedly hit *G*_1_ if a low to moderate level of noise was added to Cortex neuron activity, but performance dropped off with high levels of noise. Figure 6 illustrates, in a manner similar to Figure 3, example model neuron activity for a model run with σ = 0.1. The rest of the simulations in this paper were run with no noise.

### 3.2. Experiment 2: two simultaneous targets (*G*_*1*_ and *G*_2far_)

**Figure 7:**
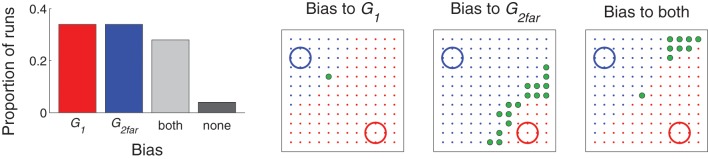
**Distribution of movements in Experiment 2: two simultaneous targets**. **Left**: Proportion of runs that were classified as being biased toward one target (more than half of the movements hit target *G*_1_, red, or *G*_2far_, blue); both targets (more than a quarter of the movements hit *G*_1_ and more than a quarter hit *G*_2far_, gray); or none (all others, black). **Right three graphs**: example movement distributions for runs that were classified as being biased toward *G*_1_, *G*_2far_, or both. Similar to Figure 4, learning was stopped (this time at the end of the 300 movements) and then movement resulting from each possible value of *G*_exp_ (represented by the spatial locations of the dots) was evaluated. Red dots indicate the location of *G*_exp_ that resulted in a movement made to *G*_1_ (red circle); blue dots indicate a movement made to *G*_2far_ (blue circle), and green dots indicate a movement made to *G*_exp_.

Movements that hit either of two targets, *G*_1_ (lower right of the workspace) or *G*_2far_ (upper left) (red and blue circles, respectively, in Figure 7), were reinforced according to Equation 1. However, the habituation term differentiated them. (The habituation term is β^*N*_*k*_−1^ in Equation 1, where *N*_*k*_ is the number of times target *k* has been hit and β = 0.825.) For example, even if *G*_1_ was hit many times, at the first time *G*_2far_ was hit, it was a novel event and thus the corresponding weights increased by a large amount.

Figure 7, left, plots the proportion of runs that were classified as either biased toward one of the targets, distributed between the two targets, or did not find a target (see figure caption for details on the classification criteria). While behavior in a majority of the runs was biased to a single target (e.g., middle two graphs of Figure 7), the model was capable of distributing movements to both targets (e.g., Figure 7, right). For runs which were biased to just one target, only a *G*_exp_ very near the un–preferred target produced a movement to that target.

### 3.3. Experiment 3: reinforce *G*_*1*_, then *G*_*2far*_, then *G*_*1*_ again

**Figure 8:**
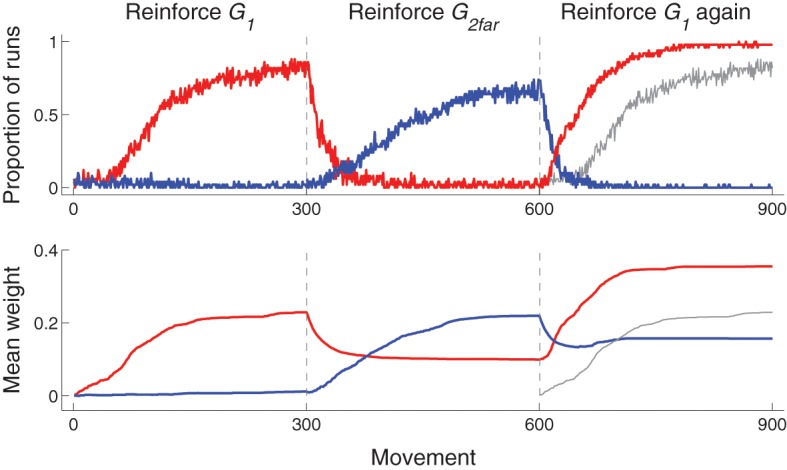
**Time-course of behavior and corticostriatal weights for Experiment 3: reinforce *G*_1_ (movements 1–300), then *G*_2far_ (301–600), then *G*_1_ again (601–900)**. **Top**: proportion of runs that hit *G*_1_ (red) or *G*_2far_ (blue) as a function of movement number. The proportion of runs that hit *G*_1_ during movements 1–300 are redrawn at horizontal positions 601–900 as a gray line for comparison of performance between initial acquisition (movements 1–300) and reacquisition (movements 601–900) of *G*_1_. **Bottom**: Mean (across runs) weight from Context neuron to the D1 neuron corresponding to most movements that hit *G*_1_ (red) or *G*_2far_ (blue) for that particular run. The D1 neuron that corresponded to most movements that hit each target was determined by finding the maximum weight from Context to D1 neurons at the end of each 300 movement segment. Because several movements can hit each target, only runs in which the same D1 neuron was selected at movement 300 and movement 900 (i.e., for movements that hit *G*_1_) were included (16 out of 50 runs were excluded). That for weights from Context neuron to D2 neurons followed a similar pattern and are not plotted. Similar to the graphs in the top row, mean weight during movements 1–300 are plotted again at movements 601–900 in gray for comparison purposes.

The use of experience-based learning rules—weight modification (Equation 1) is dependent on actual behavior—and a habituation term leads to a type of memory that can influence subsequent behavior in a changing environment. This is illustrated with experiments in which only movements to *G*_1_ are reinforced for 300 movements, then only movements to *G*_2far_ are reinforced (at which point the habituation term for *G*_1_ is reset), and then only movements to *G*_1_ are reinforced again. As shown in Figure 8, top row, which plots the proportion of runs that hit each target as a function of movement number, the reacquisition of *G*_1_ (movements 601–900) occurred faster than the initial acquisition (movements 1–300) of *G*_1_.

The enhanced acquisition is because corticostriatal weights corresponding to movements toward *G*_1_, illustrated in red in Figure 8, bottom row, increased to a stable value (of about 0.2 in the figure) during first acquisition. (The habituation prevents it from increasing any more after the target had been repeatedly hit.) During movements 301–600, *G*_2far_ was reinforced (and *G*_1_ was no longer reinforced). The model continued to move to *G*_1_ early in the second set of movements, but, because *G*_1_ was no longer reinforced, the corresponding weights decreased. As the weights decreased, the bias zone around *G*_1_ decreased and the model was free to move to other locations, including toward *G*_2far_. As a new bias zone, now centered on *G*_2far_, was established, the model stopped moving to *G*_1_. Because movements toward *G*_1_ were no longer made, weights associated with moving to *G*_1_ ceased to decrease. When movements to *G*_1_ were reinforced again, those weights were already above zero and thus *G*_1_ was reacquired faster than it was initially acquired. In addition, due to resetting the habituation term, the weights increased to a greater value than the previous high value.

This pattern of activity provides a simple mechanism that can be used to partially explain the findings that practice sessions that are separated in time lead to enhanced acquisition and performance compared to practice sessions that are massed together (Ammons, 1950; Baddeley and Longman, 1978) (though such effects do not necessarily apply to all types of tasks, e.g., Lee and Genovese 1989).

### 3.4. Experiment 4: reinforce *G*_*1*_, then either *G*_2far_ or *G*_2near_

**Figure 9:**
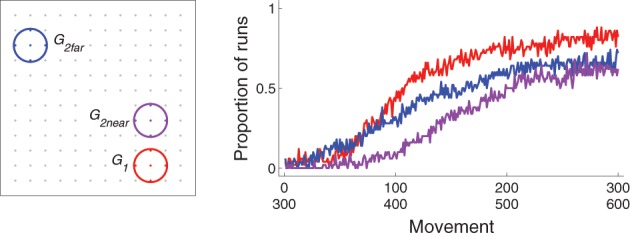
**Behavior for Experiment 4: reinforce *G*_1_ (movements 1–300) and then either *G*_2far_ or *G*_2near_ (301–600)**. **Left**: locations of the three targets in the workspace (dots indicate locations corresponding to possible values of *G*_exp_, colored gray if those locations do not lie within a target area. **Right**: proportion of runs that hit *G*_1_ for movements 1–300 or *G*_2far_ or *G*_2near_ for movements 301–600.

When one target is reinforced for a period of time, and then another is reinforced instead, how well the second reinforced target is acquired depends on its proximity to the first target. This is illustrated by comparing the results of experiments in which the second target (*G*_2far_, blue in Figure 9) was far from the first one with those in which the second target (*G*_2near_, purple) was near the first one. Figure 9 plots the proportion of runs for which the first and second targets were hit as a function of movement for the different sets. The first target (*G*_1_, red) was acquired the fastest. The far second target (*G*_2far_) was acquired faster than the near second target (*G*_2near_).

The discrepancy between acquiring the second targets is explained by the bias zone. A well-learned model has corticostriatal weights such that the bias zone is large. When the bias zone is centered around *G*_1_, un–reinforced movements to *G*_1_ must happen in order for weights to decrease, after which the bias zone shrinks and movements to other locations can be made. Movements to locations far from *G*_1_ are available earlier than movements to locations near *G*_1_ as the bias zone shrinks. Thus, a second target far from *G*_1_ will be more-easily acquired than a second target near *G*_1_.

### 3.5. Experiment 5: movement redistribution under different bias conditions

As movements made to a target increase, movements made to other locations must decrease: movements are redistributed over the workspace. The previous sections focused on movement redistribution in our model with only BG-mediated biasing (Equation 1). Here we describe metrics of movement redistribution that will allow us to compare how movements are redistributed under different bias conditions. We focus on model runs in which only movements made to one target (*G*_1_) were reinforced.

#### Redistribution metric

**Figure 10:**
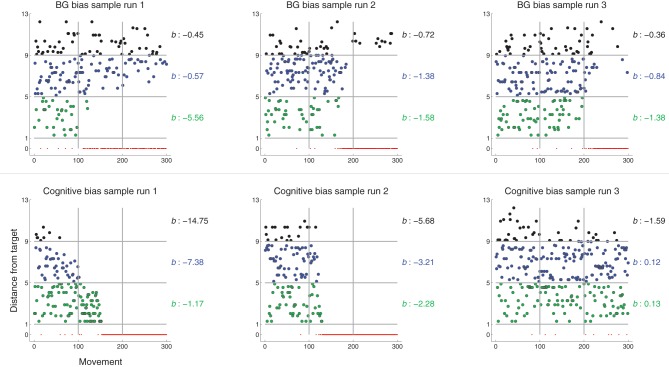
**Distance from *G*_1_ of executed movement (in units of** θ**_*G*_) as a function of movement number (similar to Figure 5, right) for sample runs of models using BG-mediated biasing (top row) or Cognitive biasing (bottom)**. Color of dot indicates the spatial zone (defined relative to target center) in which the movement lies. Horizontal lines indicate spatial zone borders. Vertical lines indicate temporal chunk borders. The parameter *b* indicates the rate of decrease of movements falling within each spatial zone. The more negative *b* is, the greater the rate of decrease (see text for details).

The expanding bias zone (Results section 3.1 and Figure 4) that results from BG-mediated biasing results in a pattern of behavior such that movements made near, but not at, the target decrease in likelihood earlier than movements made far from the target. For each run, we quantify the rate of decrease as a function of distance from target. Briefly (see Figure 10), movements that did not hit the target were coarsely categorized into three temporal chunks and three spatial zones (vertical and horizontal lines, respectively, in Figure 10). Temporal chunk one includes the first 100 movements; temporal chunk two includes the second 100 movements; and temporal chunk three includes the last 100 movements. Recalling that θ_*G*_ is target radius and letting *dX* be the distance of a movement from target center, the spatial zones are 1) θ_*G*_ < *dX* ≤ 5θ_*G*_ (green points in Figure 10), 2) 5θ_*G*_ < *dX* ≤ 9θ_*G*_ (blue), and 3) 9θ_*G*_ < *dX* (black). The number of movements that fell into spatial zone *i* from temporal chunks 1 to 2 to 3 was fit to an equation of the form *e*^*b*_*i*_(*j* − 1)^, where *j* refers to temporal chunk. The rate of decrease of the number movements was quantified by the parameter *b*_*i*_. A more negative *b*_*i*_ indicates a greater rate of decrease (see the Supplementary section for more details).

#### Movement redistribution across different bias conditions

Figure 10, top row, graphs movement distance from target as a function of movement number for three sample runs under BG-mediated bias (these graphs are similar to Figure 5, right). In all three cases, *b*_1_ < *b*_2_ < *b*_3_, i.e., the rate of decrease of movements made near but not at the target is greater than that of movements made far from the target. This is in line with the behavioral pattern we would expect given the expanding bias zone (Figure 4) that results from BG-mediated biasing. Regarding the specific sample runs in Figure 10, top row, the rate of decrease of movements from the first sample run that fell within zone one is greater than that of the second sample run, which is greater than that of the third sample run. This, also, is reflected in the *b* metrics.

The same process was used to determine *b* metrics for models that biased movement selection with different mechanisms. Recall from Methods section 2.5 that, if there is no “Cognitive bias,” movements suggested by the Explorer (*G*_exp_) were randomly selected from a uniform distribution over all possible movements. Under the Cognitive bias scheme (described in Methods section 2.5 and the Supplementary section), every time the target is hit, the set of possible movements from which *G*_exp_ is selected decreases: movements further from target center are removed from the set earlier than movements closer to target center. Movement redistribution under a Cognitive bias thus follows a trend opposite that under BG-mediated bias: *b*_1_ > *b*_2_ > *b*_3_ (Figure 10, bottom row).

For a given run of a model using BG-mediated bias, *b* for spatial zones closer to the target should be more negative than *b* for zones farther from the target. Thus, we expect *b*_3_ − *b*_2_ > 0 and *b*_2_ − *b*_1_ > 0 in models using BG-mediated bias. Models using the Cognitive bias should exhibit opposite behavior: *b*_3_ − *b*_2_ < 0 and *b*_2_ − *b*_1_ < 0. The differences should be zero if the transition from variation to repetition does not follow a structured pattern (i.e., the frequency of movements to non-target areas decreases uniformly).

**Figure 11:**
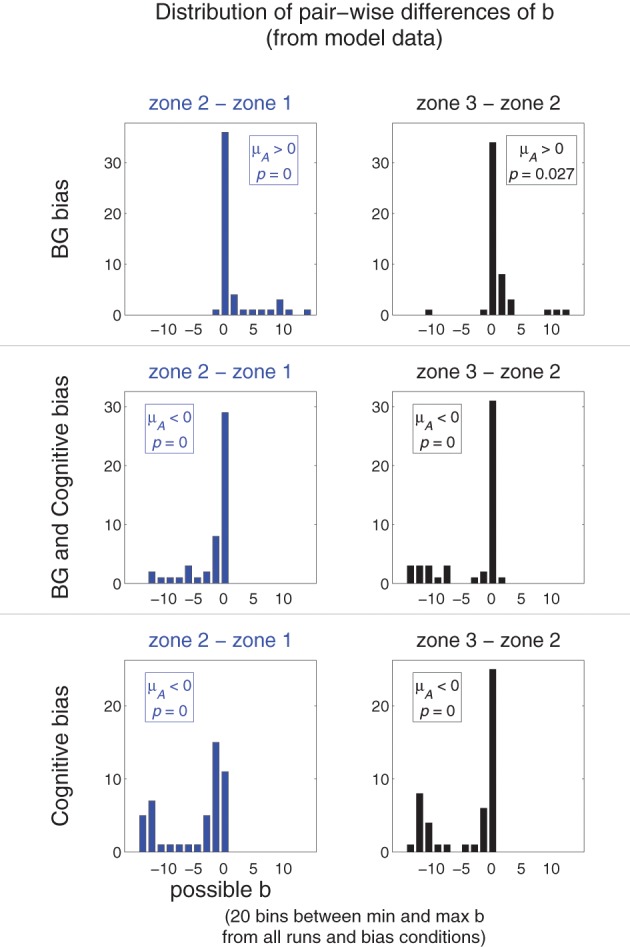
**Comparing redistribution patterns in Experiment 5: movement redistribution under different bias conditions**. Each graph is a histogram illustrating the distribution of pair-wise differences (by run) of *b* parameters of model runs using different bias conditions (arranged by row). That for *b*_3_ − *b*_2_ is in the right column (black); that for *b*_2_ − *b*_1_ is in the left column (blue). Bin widths and locations were determined as follows: the minimum and maximum *b* of all possible *b* from all analyzed runs from all conditions defined the range of possible *b*. This range was divided into 20 evenly-spaced bins of uniform size. The means of the samples of *b* parameters were tested according to the hypotheses that they have a mean μ_*A*_ > 0 or μ_*A*_ < 0 (indicated, along with *p* value, in each graph).

Figure 11 plots the distribution of pair-wise (by run) differences *b*_3_ − *b*_2_ (right column, black) and *b*_2_ − *b*_1_ (left column, blue) of model runs using different bias conditions (arranged by row). The means of the distributions were also tested against the null hypothesis that they are zero (single sample one-tailed *t*-tests). The distributions of the pair-wise differences for models using a BG bias (top row) were positive; that for models using a Cognitive bias (bottom) were negative; and that for using both biasing mechanisms (middle) were also negative (though visual inspection suggests that the Cognitive bias condition has more extreme negative pair-wise differences than does the combined bias condition). Thus, this analysis was able to capture the general trends that were seen in the different bias conditions of the model.

## 4. Discussion

As described in a recent theory of *action discovery* (Redgrave and Gurney, 2006; Redgrave et al., 2008, 2011, 2013; Gurney et al., 2013), when an unexpected sensory event occurs, animals transition from executing a variety of movements to repeating movements that may have caused the event. A transition from variation to repetition often follows non-random, structured patterns that may be explained with sophisticated cognitive mechanisms (e.g., Dearden et al. 1998; Dimitrakakis 2006; Simsek and Barto 2006). However, in action discovery, simple non-cognitive mechanisms involving dopamine modulation of basal ganglia (BG) activity are thought to play a prominent role in behavioral biasing. In this paper we use a biologically-plausible computational model to demonstrate that a structured transition from variation to repetition can emerge from processing within such simple mechanisms. Such behavior is due to the following features on which our model, unlike most previous models of BG function, focuses: (i) the BG does not bias behavior directly, but modulates cortical response to excitation (Chevalier and Deniau, 1990; Mink, 1996; Humphries and Gurney, 2002; Cohen and Frank, 2009; Redgrave et al., 2011; Baldassarre et al., 2013); (ii) excitation to cortex follows a pattern that evolves from weakly exciting all neurons to strongly exciting only one neuron (Britten et al., 1992; Platt and Glimcher, 1999; Huk and Shadlen, 2005; Bogacz et al., 2006; Gold and Shadlen, 2007; Lepora et al., 2012). By including these features in our model, we show that sophisticated cognitive mechanisms may not always be necessary to develop a structured transition from variation to repetition.

In our model, movements occur by selecting an end-point (spatial location) to which to move. Movements that terminated in a target area were reinforced so that the selection of such end-points increased in frequency. The transition from executing a variety of movements to executing just the reinforced movements followed a structured pattern: as end-points at the target location increased in frequency, end-points near, but not at, the target location decreased in frequency at a greater rate than end-points far from the target. We refer to the area around the target area in which end-point frequency decreased as a “bias zone” (Figures 4, 10, top), and the bias zone increased in size as the target was repeatedly hit. The graded shift from variation (a small bias zone) to repetition (a large bias zone) allows for the discovery of a second target area in some cases (Figure 7), and also results in specific patterns of behavior if the target area moves (Figures 8, 9).

In addition, in action discovery, phasic DA activity in response to achievement of the outcome (e.g., hitting the reinforced target area) decreases as associative brain areas learn to predict the outcome's occurrence (Redgrave and Gurney, 2006; Redgrave et al., 2008, 2011, 2013; Gurney et al., 2013; Mirolli et al., 2013). This may be thought of as a type of intrinsic motivation (IM) in that the outcome need not have hedonic value in order to be reinforcing (Oudeyer and Kaplan, 2007; Baldassarre, 2011; Barto, 2013; Barto et al., 2013; Gottlieb et al., 2013; Gurney et al., 2013). The type of IM in action discovery is best described as some combination of novelty and surprise (Barto et al., 2013). A detailed account of exactly how the prediction process may be implemented in the brain is beyond the scope of this paper. We mimic its effects in our model with a simple habituation mechanism similar that used in neural network models of novelty detection (Marsland, 2009). Here, the reinforcing effects of an outcome with which the model has little recent experience is greater than the reinforcing effects of an outcome with which the model has much recent experience. The habituation term (β^*N*_*k*_−1^ in equation 1) influences behavioral patterns, particularly in tasks in which more than one target area is reinforced (Figure 7) or the target area changes (Figures 8, 9). Unlike the reward prediction error hypothesis of phasic DA neuron activity (Houk et al., 1995; Schultz et al., 1997), habituation is a mechanism that does not rely on extrinsic motivation by which phasic DA neuron activity, and hence rate of change of the rate of corticostriatal plasticity, decreases with continued occurrences of the outcome.

We also implement models in which a structured transition from variation to repetition is that which would be expected if one type of more sophisticated mechanism (“Cognitive biasing”) is in effect. The pattern of behavior (Figure 10, bottom) is then different than that of BG-only biasing. Finally, we have devised a method for capturing such differences with quantitative measures (Figures 10, 11) which will allow us to make contact with future behavioral experiments investigating how different brain areas contribute to biasing behavior in tasks similar to model tasks. In continuing work, we are devising such behavioral experiments. Preliminary results suggest that our quantitative measure will allow us to compare the effects of different biasing mechanisms by examining behavior from different systems (e.g., model versus human), different workspaces, different target sizes, and different target locations, etc. Possible mechanisms by which to isolate different brain mechanisms include explicit instructions, use of different stimuli (Thirkettle et al., 2013b), or use of distractor tasks (Stocco et al., 2009).

As with any computational model of brain systems, the mechanisms described in this paper should be viewed as being a part of a complex system of interacting parts. We've isolated the effects of the specific mechanisms we've investigated in order to demonstrate how a structured transition from variation to repetition can emerge from those mechanisms. In the next subsection we discuss the implications of some of these choices in greater detail and how to expand on them to include more sophisticated systems.

### 4.1. A multi-stage selection process

Recall that, for each movement in our model, the pattern of excitation from “Explorer” to “Cortex” evolves from weakly-exciting all neurons to strongly-exciting one neuron (referred to as *G*_exp_, the focus of excitation). The weak excitation of all neurons early in the evolution allows for corticostriatal plasticity to bias behavior. Behavior can also be biased by the choice of *G*_exp_, the effects of which are greater later in the evolution. Thus, the evolving excitation pattern from Explorer to Cortex allows for a multi-stage selection process. We expand on these points below.

Through corticostriatal plasticity and BG selection mechanisms, Cortex neurons that are only weakly excited during the early stages of excitation from Explorer can increase in activity at a greater rate than other Cortex neurons. BG selection mechanisms also enable these neurons to suppress the responses of other Cortex neurons to subsequent strong excitation (e.g., Figure 3). The expanding bias zone (described in Results section 3.1 and Figure 4) that is seen in models using BG-mediated biasing emerges from the pattern of excitation from Explorer to Cortex. Because the model task was a spatial reaching task, a topographic representation was used that revealed an apparent dependency between movements: neurons in Explorer near the focus of excitation (*G*_exp_) were excited more than neurons far from the focus.

However, a different pattern may be revealed in other types of tasks. In general, the pattern of activity is likely to be influenced by perceptual processing of sensory information. For example, the theory of affordances (Gibson, 1977, 1986) suggests that the perception of objects preferentially primes neurons that correspond to actions that can operate on those objects, e.g., the perception of a mug would prime a grasping action. Thus, the pattern of excitation in these conditions would preferentially excite those neurons, and excitation may follow a pattern that is different than the one used in this paper. Because BG modulates how Cortex responds to excitation rather than directly-exciting movements, any behavioral pattern controlled by BG-mediated biasing would depend on the pattern of excitation to Cortex. Thus, different patterns of exploration, and different patterns of a structured transition from variation to repetition, would be observed in different environments and tasks.

We envision that more sophisticated mechanisms (e.g., our Cognitive biasing) can be expressed in our model in the later part of the evolving excitation pattern of the Explorer, i.e., in how *G*_exp_ is chosen. One such mechanism may search the workspace in a way that is more intelligent than random, such as a spiral or raster-like search pattern that does not repeat itself until all possible movements have been executed. The choice of *G*_exp_ could also be adaptive, including using mechanisms by which a transition from variation to repetition is governed by mechanisms based on measures of optimality, uncertainty, or other task-related variables (Dearden et al., 1998; Daw et al., 2006; Dimitrakakis, 2006; Simsek and Barto, 2006; Cohen et al., 2007).

Thus, the early part of the evolving excitation pattern from Explorer to Cortex comprises weak excitation that is influenced by perception of the environment (e.g., affordances or, in our model, possible movement locations) or simple mechanisms. The later part of the evolution allows for more complicated mechanisms that may require more processing time to also influence behavior. We have focused mostly on simple mechanisms in this paper, but the evolving pattern of excitation can be used to implement proposed theories that focus on multiple influences on behavior, e.g., Kawato (1990); Rosenstein and Barto (2004); Daw et al. (2005); Shah and Barto (2009).

### 4.2. Action discovery with complicated behaviors

There are many types of movements or behaviors that can affect the environment, e.g., making a gesture (regardless of spatial location), manipulating objects in the environment, or making a sequence of movements. In this paper we focused on a simple type of action in which the system, able to select a spatial end-point of movement, must discover the end-point(s) that delivers an outcome. On a more abstract level, this is similar to “*n*-armed bandit” problems, in which the system must discover which out of a set of *n* actions is followed by the most rewarding consequences in a one-step decision task (e.g., Sutton and Barto 1998). The general process of action discovery (Redgrave and Gurney, 2006; Redgrave et al., 2008, 2011, 2013; Gurney et al., 2013) is also concerned with discovering the temporal and structural components of a complex behavior that affects the environment. These problems are similar to the those of temporal and structural credit assignment problems (Minksy, 1961; Sutton, 1984, 1988; Barto, 1985; Sutton and Barto, 1998), which we briefly describe below.

One form of the temporal credit assignment problem is exposed in systems in which a series of actions is required in order to achieve an outcome, and there is great redundancy: a large number of different (but possibly overlapping) sequences can achieve the outcome. How does the agent discover the most direct sequence, i.e., the sequence that uses the fewest actions? This redundancy is often resolved by assigning a cost for each executed action and using optimal control methods to achieve the goal while also minimizing cost (e.g., Sutton and Barto 1998). However, optimal control methods, which are designed to find behavior that minimizes cost according to an arbitrary cost function, may use mechanisms that are more sophisticated and complicated than those thought to underly action discovery. Recent modeling work (Shah and Gurney, 2011; Chersi et al., 2013) has shown that a simpler learning rule that does not incorporate cost per action can discover the most direct sequence of actions in a redundant system. Such behavior remains stable for a period of time, but, if learning is not attenuated, extraneous actions are incorporated with extended experience (Shah and Gurney, 2011).

The structural credit assignment problem is exposed when a system can execute many actions simultaneously and the outcome depends only on the simultaneous execution of a small subset of those. When behavior is composed of several components, and the outcome is contingent on only some of those components, variation allows the animal to determine which components are relevant and to “weed out” the irrelevant components. We have not addressed this problem directly, but previous work on the structural credit assignment problem in RL offers promising directions (Barto and Sutton, 1981; Barto et al., 1981; Barto, 1985; Barto and Anandan, 1985; Gullapalli, 1990).

### 4.3. Conclusion

How biasing causes a transition from variation to repetition so as to converge on the specific movements that cause an outcome is a fundamental problem in the process of action discovery. With a simple model of a restricted aspect of action discovery, which includes neural processing features not included in most other models of BG function, we are able to describe the effects of different types of behavioral biasing. The results reported in this paper describe a first step in understanding the more processes at work in general action discovery.

### Conflict of interest statement

The authors declare that the research was conducted in the absence of any commercial or financial relationships that could be construed as a potential conflict of interest.
