# Supplementary material for: Emergent structured transition from variation to repetition in a biologically-plausible model of learning in basal ganglia
Source: Front Psychol. 2014 Feb 11;5:91. doi: 10.3389/fpsyg.2014.00091 (PMC3920096; doi:10.3389/fpsyg.2014.00091)
Supplement: Supplementary file 1 [file DataSheet1.PDF]

SUPPLEMENTARY MATERIAL FOR

Emergent structured transition from variation to repetition in a biologically-plausible model of learning in basal ganglia.

Frontiers in Cognitive Science

Ashvin Shah and Kevin Gurney
Department of Psychology, The University of Sheffield, Western Bank, Sheffield, United Kingdom, S10 2TP

This article is part of the research topic *Intrinsic motivations and open-ended development in animals, humans, and robots*

1 Supplementary Section I: Model Details

1.1 Explorer

The entire hand-crafted evolution period of the Explorer takes T_E (= 750) time steps, which is the length of a model trial. Letting E_i be the activity of Explorer neuron i, first all E_i are set to zero. Neuron G_{exp} from the middle 12×12 section of the 14×14 grid (i.e., excluding a 1-element border) is randomly chosen from a uniform distribution over all 144 neurons. To quantify the spatial distance between each neuron i and G_{exp}, the Euclidean distance between the two is calculated and multiplied by 0.1. A random number chosen from the uniform distribution between -0.05 and 0.05 is added to this number, and the result is represented as d_i. The set of all d_i is then normalized to be between 0 and 1: $\forall\ i$, $d'_i = d_i - \min(d_i)$, and then $\forall$ i, $d''_i = d'_i/\max(d'_i)$. For the first $2T_E/3$ time steps, E_i for each i is determined by a Gaussian function centered on G_{exp} with a standard deviation (σ) that decreases as time increases:

$$\begin{aligned} \sigma(t) =&\ \tfrac{15\,(2T_E/3-t)}{2T_E/3} + 0.001 \\ s_i =&\ e^{(-d''_i/2\sigma(t))^2} \\ E_i \leftarrow&\ \left(\tfrac{t}{2T_E/3}\right) s_i\ , \end{aligned}$$

where t ranges from one to $2T_E/3$. For the final $T_E/3$ time steps, E_i = one for $i = G_{exp}$ and zero for $i \neq G_{exp}$. An example of this evolution is illustrated in Figure 2 in the main text.

This excitation pattern results in apparent dependencies between movements. Such dependencies can also be caused by Gaussian projection patterns; we confine dependencies to the excitation pattern so as to introduce fewer assumptions to model architecture.

1.2 General neural framework

All neurons in the model are leaky-integrator firing-rate neurons and follow the same general dynamics. (Gurney et al. 2001b discusses this general framework in detail.) The rate of change of the internal activation level of a neuron is defined as

$$\frac{\mathrm{d}a_i}{\mathrm{d}t} = -k(a_i - u_i),$$

where a_i is the internal activation level of neuron i, u_i is the input to neuron i, $\mathrm{d}t$ (= 0.001) is a time-step, and k is a rate constant determined by

$$k = e^{-\mathrm{d}t/\tau},$$

where $\tau = 0.01$ for Cortex neurons, $\tau = 0.005$ for Thalamus neurons, and $\tau = 0.02$ for BG neurons (grey boxes in Figure 1 of the main text).

Internal activation level a_i is interpreted as the membrane potential of neuron i near the axon hillock. The output activation of neuron i is y_i, which is a function of a_i and is interpreted as the firing-rate of neuron i:

$$y_i = \begin{cases} 0 & \text{if} \quad a_i < \epsilon \\ m(a_i - \epsilon) & \text{if} \quad a_i \leq \epsilon \leq 1/m + \epsilon \\ 1 & \text{if} \quad a > 1/m + \epsilon \end{cases},$$

where m (= 1 for all neurons) is the slope of the output function and ϵ is a threshold set to 0 for Cortex and Thalamus neurons, 0.13 for D1 and D2 neurons, -0.225 for STN neurons, -0.2 for GPe neurons, and -0.3 for SNr neurons. These parameter values are roughly similar to those used in previous models (Gurney et al., 2001a,b; Humphries and Gurney, 2002).

1.3 Cortex and Thalamus

The input, u_i^M, to Cortex neuron i consists of excitatory projections from y_i^E, the output activity of Explorer neuron i, and y_i^T, the output activity of Thalamus neuron i:

$$u_i^M = w^{E-M} y_i^E + w^{T-M} y_i^T,$$

where $w^{E-M} = 0.15$ is the magnitude of the weight of Explorer neuron i to Cortex neuron i and $w^{T-M} = 1.45$ is the magnitude of the weight of Thalamus neuron i to Cortex neuron i.

The input, u_i^T, to Thalamus neuron i consists of excitatory projections from y_i^M, the output activity of Cortex neuron i, and inhibitory input from y_i^{SNr}, the ouput of SNr neuron i:

$$u_i^T = (w^{M-T} y_i^M)[1 - w^{SNr-M} y_i^{SNr}]^+,$$

where $w^{M-T} = 1.45$ is the magnitude of the weight from Cortex neuron i to Thalamus neuron i and $w^{SNr-T} = 3$ is the magnitude of the weight from SNr neuron i to Thalamus neuron i. Note that because w^{SNr-M} is absolute magnitude, it is a non-negative scalar. The inhibitory nature of SNr projections is captured by the "minus" sign in the above equation. Also, $[x]^+$ returns 0 if $x < 0$ and x otherwise.

1.4 Basal Ganglia

Striatum (D1 and D2)

D1 represents D1-like receptor containing striatal neurons. The input, u_i^{D1}, to D1 neuron i consists of projections from y_i^M, the output activity of Cortex neuron i,

and y^C, the output activity of the Context neuron. Note that, because there is only one context in this model, Context consists of a single neuron with a constant output activity set to $y^C = 0.45$. The inputs to each D1 neuron are scaled by a multiplicative factor $(1 + \lambda_{D1})$ that represents tonic dopamine (DA) levels:

$$u_i^{D1} = (1 + \lambda_{D1})(w_i^{C-D1} y^C + w_i^{M-D1} y_i^M),$$

where w_i^{C-D1} is the weight from the Context neuron to D1 neuron i and w_i^{M-D1} is the weight from Cortex neuron i to D1 neuron i. These projections are plastic (learning rules to be described below) and thus the weights contain an explicit index i. The initial values of all w_i^{C-D1} are 0, and the initial values of all w_i^{M-D1} are 0.5. Also, tonic DA ($\lambda_{D1} = 0.2$), kept constant in our model, is not to be confused with phasic DA, on which learning depends (Grace et al., 2007).

D2 represents D2-like receptor containing striatal neurons. D2 recieves the same input projections as D1, but the effect of tonic DA is different:

$$u_i^{D2} = (1 - \lambda_{D2})(w_i^{C-D2} y^C + w_i^{M-D2} y_i^M),$$

where w_i^{C-D2} is the weight from the Context neuron to D2 neuron i and w_i^{M-D2} is the weight from Cortex neuron i to D2 neuron i. These weights are also plastic and have the initial values of 0 and 0.5, respectively, and tonic DA ($\lambda_{D2} = 0.2$) is kept constant.

Subthalamic Nucleus (STN)

STN represents the subthalamic nucleus. Similar to D1 and D2 neurons, the input, u_i^{STN} of STN neuron i consists of excitatory projections from y_i^M and y^C. It also recieves inhibitory projections from GPe:

$$u_i^{STN} = w^{C-STN} y^C + w^{M-STN} y_i^M - w^{GPe-STN} y_i^{GPe},$$

where w^{C-STN} ($= 0.04$) is the weight from the Context neuron to STN neuron i, w^{M-STN} ($= 0.15$) is the weight from Cortex neuron i to STN neuron i, and $w^{GPe-STN}$ ($= 0.15$) is the weight from GPe neuron i to STN neuron i. Unlike projections from Context and Cortex to D1 and D2, projections to STN are not plastic in this model.

Globus Pallidus (GPe)

GPe represents the external segment of the globus pallidus. GPe neuron i recieves inhibitory input from D2 neuron i and *diffuse* (i.e., one-to-all) excitatory projections from STN:

$$u_i^{GPe} = -w^{D2-GPe} y_i^{D2} + \sum_{j=1}^{N_{STN}} w_j^{STN-GPe} y_j^{STN},$$

where w^{D2-GPe} (= 0.45) is the weight of the projection from D2 neuron i to GPe neuron i and $w_j^{STN-GPe}$ (= 0.1) is the weight of the projection from STN neuron j to GPe neuron i; $w_j^{STN-GPe}$ is the same for all j. Also, $N_{STN} = 196$ is the number of neurons in STN (and all groups of neurons except for Context).

Substantia Nigra (SNr)

SNr (substantia nigra pars reticulata) neuron i recieves inhibitory input from D1 neuron i, inhibitory input from GPe neuron i, and diffuse excitatory projections from STN:

$$u_i^{SNr} = -w^{D1-SNr} y_i^{D1} - w^{GPe-SNr} y_i^{GPe} + \sum_{j=1}^{N_{STN}} w_j^{STN-SNr} y_j^{SNr},$$

where w^{D1-SNr} (= 0.45) is the weight of the projection from D1 neuron i to SNr neuron i, $w^{GPe-SNr}$ (= 0.3) is the weight of the projection from GPe neuron i to SNr neuron i, and $w_j^{STN-SNr}$ (= 0.3) is the weight of the projection from STN neuron j to SNr neuron i.

1.5 From Cortex output activity to behavior

Movement is a function of the output activities of Cortex neurons. Each Cortex neuron with an output activity above $\eta = 0.6$ "votes," with a weight proportional to its output activity level, to move towards the spatial location that corresponds to its location within the grid of neurons. At time step t, the end-point to which to move is:

$$X_M(t) = \frac{\sum_{i \in M} x_{M_i}(t)\ [y_i^M(t)]^\eta}{\sum_{i \in M} [y_i^M(t)]^\eta},$$

where y_i^M is the output activity of Cortex neuron i, $[y_i^M]^\eta$ returns zero if $y_i^M < \eta$ and y_i^M otherwise, and x_{M_i} represents the preferred spatial location of Cortex neuron i. In most cases, because of the selection properties of the BG, the output activity of only one Cortex neuron rises above η. At each time step, if any $y_i^M > \eta$, a simple plant causes a movement from the current position, x_p, toward X_M:

$$\Delta x_p(t) = \tau(X_m(t) - x_p(t-1)),$$

where $\tau = 0.9$. Any learning is based on $x_p(T_E)$, the position at time T_E.

1.6 Biasing of behavior in the model

BG-mediated biasing

Corticostriatal weights (w^{C-D1}, w^{M-D1}, w^{C-D2}, and w^{M-D2}) are subject to dopamine-dependent plasticity (Wickens, 2009; Calabresi et al., 2007). If the target is hit, corticostriatal weights are increased according to the following Hebbian-like rules:

$$\begin{aligned}\Delta w_i^{C-D1} &= \alpha\, \beta^{N_k - 1}\, y_i^{D1}\, (W_{max} - w_i^{C-D1}) \\ \Delta w_i^{M-D1} &= \alpha\, \beta^{N_k - 1}\, y_i^{D1}\, y_i^{M}\, (W_{max} - w_i^{M-D1})\end{aligned}$$

where $\alpha = 0.1$ is a step-size, $W_{max} = 1$ is the maximum strength of a synapse, $\beta = 0.825$ is a novelty discount (Marsland, 2009), and N_k is the number of times target k has been hit. Because there is only one Context neuron, its constant output activity (y_i^C) is kept out of the weight update equation for w^{C-D1}. The same type of rules dictate updates for w^{C-D2} and w^{M-D2}.

If a target is not hit, corticostriatal weights are decreased:

$$\begin{aligned}\Delta w_i^{C-D1} &= -\alpha_d\, y_i^{D1} \\ \Delta w_i^{M-D1} &= -\alpha_d\, y_i^{D1}\, y_i^{M}\end{aligned}$$

where $\alpha_d = 0.1\alpha$ is the step-size (again, the same type of rules dictate updates for w^{C-D2} and w^{M-D2}). In addition, a lower bound is imposed: any w_i^{C-D1} or w_i^{C-D2} below -0.1 is set to -0.1, and any w_i^{M-D1} or w_i^{M-D2} below 0 is set to 0.

Cognitive biasing

"Cognitive biasing" in our model is a simple hand-crafted form of biasing that "zooms in" on the target as the target is repeatedly hit. As implemented here, the largest distance, D_G^{max}, between X_G (the location of the center of the target) and the border of the workspace is calculated. This defines an imaginary square, S_G, with borders of length $2D_G^{max}$, centered on X_G. Unless X_G is at the exact center of the workspace, a portion of S_G will reside outside the workspace. G_{exp} is chosen randomly from the intersection of the workspace and S_G; before the target is hit, this intersection is the entire workspace. Each time the target is hit, the outermost border of S_G is removed until only locations within θ_G (target radius) of X_G remain.

2 Supplementary Section II: Defining the b Metric

Movements were coarsely catagorized as belonging to one of three temporal chunks and one of three spatial zones (e.g., vertical and horizontal lines, respectively, in Figure 9 of the main text). For each run, the number of movements, $N(i,j)$, that

were catagorized as belonging to spatial zone i and temporal chunk j were counted. For each spatial zone, these numbers were divided by the maximum number across the temporal chunks, i.e., for each i and j,

$$n(i,j) = \frac{N(i,j)}{\max_j N(i,j)},$$

where $\max_j N(i,j)$ returns the maximum $N(i,j)$ across all j. Thus, $0 \leq n(i,j) \leq 1$, and the maximum (across all j) $n(i,j)$ is one. This normalization allows us to compare the rate of change of number of movements made into a particular spatial zone as a function of temporal chunk between the different spatial zones and runs. To prevent division by zero, runs that made zero movements to a spatial zone were excluded from analysis.

For each spatial zone (i), $n(i,j)$ at $j = 1$, 2, and 3 (for the three temporal chunks) was fit to $a\,\mathrm{e}^{b_i(j-1)}$ using the Matlab function *lsqcurvefit* (Matlab Optimization Toolbox R2011, MathWorks, Natick, Massachusetts, USA). The parameter b_i of this equation quantified the rate of decrease of the number of movements made to each spatial zone as a function of temporal chunk: a more negative b_i indicates a greater rate of decrease.

References

Calabresi, P., Picconi, B., Tozzi, A., and DiFilippo, M. (2007). Dopamine-mediated regulation of corticostriatal synaptic plasticity. *Trends in Neuroscience*, 30:211–219.

Grace, A., Floresco, S., Goto, Y., and Lodge, D. (2007). Regulation of firing of dopaminergic neurons and control of goal-directed behaviors. *Trends in Neuroscience*, 30:220–227.

Gurney, K., Prescott, T., and Redgrave, R. (2001a). A computational model of action selection in the basal ganglia. I. A new functional anatomy. *Biological Cybernetics*, 84:401–410.

Gurney, K., Redgrave, R., and Prescott, T. (2001b). A computational model of action selection in the basal ganglia. II. Analysis and simulation of behaviour. *Biological Cybernetics*, 84:411–423.

Humphries, M. and Gurney, K. (2002). The role of intra-thalamic and thalamocortical circuits in action selection. *Network: Computation in Neural Systems*, 13:131–156.

Marsland, S. (2009). Using habituation in machine learning. *Neurobiology of learning and memory*, 92:260–266.

Wickens, J. R. (2009). Synaptic plasticity in the basal ganglia. *Behavioural Brain Research*, 199:119–128.
